# Effect of Short-Term Low-Nitrogen Addition on Carbon, Nitrogen and Phosphorus of Vegetation-Soil in Alpine Meadow

**DOI:** 10.3390/ijerph182010998

**Published:** 2021-10-19

**Authors:** Zhen’an Yang, Wei Zhan, Lin Jiang, Huai Chen

**Affiliations:** 1 Key Laboratory of Southwest China Wildlife Resources Conservation, China West Normal University, Nanchong 637009, China; 2 Key Laboratory of Mountain Ecological Restoration and Bioresource Utilization & Ecological Restoration and Biodiversity Conservation Key Laboratory of Sichuan Province, Chengdu Institute of Biology, Chinese Academy of Sciences, Chengdu 610041, China; zhanwei0125@gmail.com; 3 Institute of Environment and Ecology, Shandong Normal University, Ji’nan 250358, China; jianglinlm@126.com

**Keywords:** community, rhizosphere, bulk, carbon, nutrient, stoichiometry, linkage, Qinghai-Tibetan Plateau

## Abstract

As one of the nitrogen (N) limitation ecosystems, alpine meadows have significant effects on their structure and function. However, research on the response and linkage of vegetation-soil to short-term low-level N deposition with rhizosphere processes is scant. We conducted a four level N addition (0, 20, 40, and 80 kg N ha^−^^1^ y^−1^) field experiment in an alpine meadow on the Qinghai-Tibetan Plateau (QTP) from July 2014 to August 2016. We analyzed the community characteristics, vegetation (shoots and roots), total carbon (TC), nutrients, soil (rhizosphere and bulk) properties, and the linkage between vegetation and soil under different N addition rates. Our results showed that (i) N addition significantly increased and decreased the concentration of soil nitrate nitrogen (NO_3_^−^-N) and ammonium nitrogen, and the soil pH, respectively; (ii) there were significant correlations between soil (rhizosphere and bulk) NO_3_^−^-N and total nitrogen (TN), and root TN, and there was no strong correlation between plant and soil TC, TN and total phosphorus, and their stoichiometry under different N addition rates. The results suggest that short-term low-N addition affected the plant community, vegetation, and soil TC, TN, TP, and their stoichiometry insignificantly, and that the correlation between plant and soil TC, TN, and TP, and their stoichiometry were insignificant.

## 1. Introduction

As an important feature of global change, anthropogenic nitrogen (N) deposition has increased significantly since the Industrial Revolution [1]. Previous studies show that global atmospheric N deposition has increased two-fold over the last century and will increase further in most regions, in the next decades [2]. The majority of studies on the topic have conducted that N additions have altered the structure and function of terrestrial ecosystems such as forests [3], alpine meadows [4,5,6,7,8,9,10], alpine shrublands [2], grasslands [1,9,11,12], and swamps [13] with the method of field N addition experiments [1,2,3,8,9] and meta-analysis [8,14,15]. For instance, increased N deposition could decrease the diversity of species partly caused by the inhibition or extinction of the growth of vulnerable or rare species [6,10] and increase primary productivity [1,10], carbon and nutrient storage capacity in terrestrial ecosystems [2,9]. 

Vegetation and soil are considered important, and relatively independent parts of terrestrial ecosystems [16], which influence or even alter the structure and function of terrestrial ecosystems, through a series of carbon (C) and nutrient cycling [17,18,19]. Specifically, vegetation releases the fixed carbon into soil, as the substrates of soil microorganisms, through litter and rhizodeposition (sloughed-off root cap, border cells, mucilage, and exudate) [20]. Simultaneously, through the absorption of roots and mycorrhizal fungi, plants absorb nutrients such as nitrogen (N) and phosphorus (P) from the decomposition of organic matter in the soil, which are then transported to the aboveground part [18,21]. Therefore, many related studies show that the C, N, and P contents and their stoichiometric ratios of vegetation and soil are highly coupled, as they are cycle in the same system [18,22,23,24]. For example, although there are differences in the acquisition strategies of nutrients and the use efficiencies of understory plants and overstory trees, there is a significant correlation between N and P contents and the N:P ratio of plant and soil on the Loess Plateau, China [23]. 

Moreover, according to numerous studies, the rhizosphere is not only a micro-area that is significantly affected by the root activity with a characteristic of nutrient accumulation, higher microbial biomass, and activity, but also the most powerful tool to ascertain the nutrient transport, flow, and cycling of terrestrial ecosystems [2,18,20,25,26]. For example, different grazing intensities (0, 0.7, 1.2, and 1.6 yaks ha^−^^1^) had an insignificant effect on the enrichment ratios of the C and nutrients rhizosphere soil (RS) in an alpine meadow on the eastern Qinghai-Tibetan Plateau (QTP) [26], suggesting that the rhizosphere restricts the transport and flow of nutrients between the soil and plants, to ensure the stability of the ecosystem [21,26]. Increasingly, studies are examining the responses of rhizosphere processes to N addition. However, research on how N addition affects the linkage of above- and below-ground processes is scant [2].

The QTP is a relatively fragile system worldwide [2,26] which is threatened by the increasing rate of natural N deposition (8.7–13.8 kg ha^−^^1^ y^−1^) [10,27]. As the dominant type of vegetation (more than 40%) on the QTP, alpine meadow was not only a fundamental source of livelihood of local residents, but also a significant ecological barrier for downstream populations [26]. However, this vegetation is usually limited by soil N owing to the slow N mineralization rates [6,10,28]. A number of studies have focused on the responses of alpine meadows on the QTP to N addition [2,4,5,6,7,8,9,10]. For example, a 3-year low-level N addition study (0, 10, 20, 40, and 80 kg N ha^−1^ y^−1^) in alpine meadows on the QTP suggested that compared to soil properties, plant community characteristics (cover and biomass) are more sensitive to increasing N inputs [10]. Further, a study on a 4-year low-level N addition study (0, 10, 20, and 40 kg N ha^−1^ y^−1^) indicated that increasing atmospheric N deposition is not conducive to soil organic carbon pool accumulation in the alpine meadow of Haibei on the QTP [5]. However, the research on the response of vegetation-soil to short-term low-level N deposition with rhizosphere processes is scant.

We conducted a field experiment using four rates of N addition (0, 20, 40, and 80 kg N ha^−1^ y^−1^) from July 2014 to August 2016 in Hongyuan County, in the northwest of Sichuan Province on the northeastern margin of the QTP. We surveyed the community characteristics (cover, species number, above- and belowground biomass, and shoot/root), vegetation (shoots and roots), and soil C, N, and P content and their stoichiometry. This study aims to (1) clarify the effects of N addition on vegetation, (2) examine the soil (rhizosphere and bulk) properties to ascertain the coupled changes in the rhizosphere and bulk soil processes under N addition, and (3) understand the linkage of vegetation (shoots and roots) and soil (rhizosphere and bulk) C and nutrients under N addition.

## 2. Materials and Methods

### 2.1. Study Area and the Experimental Design

**Figure 1:**
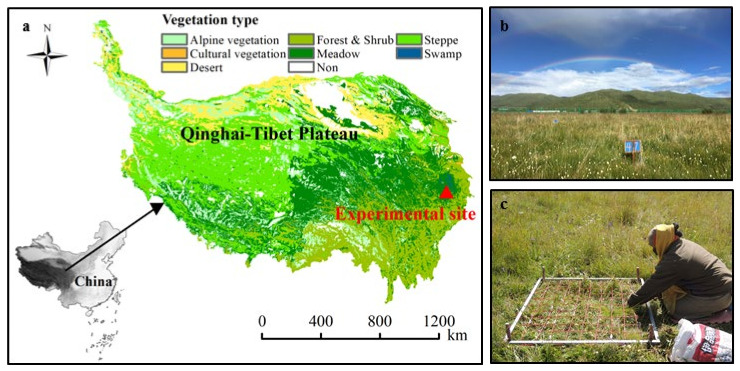
Location (**a**), sample plot (**b**) and vegetation investigation (**c**) of study area in Qinghai-Tibetan Plateau.

This study was conducted in a typical alpine meadow in Hongyuan County, in the northeast of the QTP, China (latitude 32°57’56″ N, longitude 102°36’56″ E and 3480 m a.s.l.; Figure 1). The mean annual temperature and precipitation in this area from 1961 to 2015, were 1.57 °C and 751.21 mm, respectively (data from Chinese Meteorological Data Service Centre, http://data.cma.cn//, accessed on 20 March 2021). This area, which was previously used as a winter pasture for local herders to raise yaks, was enclosed in May 2014. The main species in this area are *Elymus nutans, Deschampisa caespitosa, Kobresia setchwanensis, Saussurea nigrescens, Trollius farreri, Anemone rivularis,* and *Astragalus polycladus*.

According to the natural N deposition of the QTP, which is 8.7–13.8 kg ha^−^^1^ y^−1^ [10,27], we set 4 N addition rates (0, 20, 40, and 80 kg N ha^−^^1^ y^−1^), and selected the NH_4_Cl (nitrogen content of 26%) as the N source, as ammonium nitrogen (NH_4_^+^-N) is the main form of natural N deposition [29,30]. Table 1 shows the experiment design. We set 16 sample plots (8 m × 8 m, 4 replicates per N addition gradient) with a wide buffer of 1 m between each plot in July 2014. Moreover, all the sample sites were designed completely randomly. The annual amount of N application was divided into five equal parts before N addition. We added the N at the beginning of each month during the growing season (May to September), as (i) it is generally believed that the growing period of the QTP is May to September, and that more than 80% of the precipitation in this area is concentrated during the growing season [26,31]; (ii) N addition during the growing season can reduce loss (leaching, volatilization, etc.), which is caused by the failure of plants to make timely use of the added N [6]. When N was added, the weighed NH_4_Cl was dissolved in 20 L of water and sprayed uniformly with a sprayer; the 20 L of water was sprayed with 0 kg N ha^−^^1^ y^−1^ (N_0_).

### 2.2. Sampling and Measurement

In early August 2016 (approximately 10 days before N addition in August, N was added for 10 months), we selected three of the four repeated treatments of each N addition treatment with good vegetation growth and those that were free of disturbance by rodents, such as Plateau pika and Plateau zokor (Figure 1b). Before sampling, we randomly selected three quadrats (1 m × 1 m) in each sample plot (8 m × 8 m), to investigate the community cover and species number. Further, each treatment comprised nine quadrats (1 m × 1 m) used to calculate covers and the number of species (Figure 1c). Then small quadrats (0.5 m × 0.5 m) were further selected in each quadrat (1 m × 1 m), to harvest the vegetation along the soil surface. Each treatment comprised nine quadrats (0.5 m × 0.5 m) with which to calculate the aboveground biomass. In each sample plot (8 m × 8 m), we randomly selected five areas (in/near the small quadrats) that could represent the vegetation distribution of each treatment, removed the dead leaves from the ground and dug up the soil samples (0.2 m × 0.2 m × 0.15 m). From each treatment, 15 soil samples were collected, to harvest plant and soil (rhizosphere and bulk) samples [26]. We then transported the soil samples to the laboratory to collect the soil samples (rhizosphere and bulk) by shaking the plants [25,26]. Specifically, we defined bulk soil (BS) as the soil that does not adhere to the roots tightly (was gently shaken down), whereas the RS is the soil attached to the roots (still attached to the roots after shaking the BS, and collected with a fine brush) [25,26]. All the plants were then separated into shoots (aboveground parts) and roots (belowground parts) along the root collar [26]. The root shoot ratio (R/S) was expressed as the ratio of belowground biomass to aboveground biomass.

We sieved all the fresh soil (rhizosphere and bulk) using a 2-mm mesh. One portion of the soil was stored at −20 °C for determination of dissolved organic carbon (DOC) and inorganic N (in the form of nitrate nitrogen (NO_3_^−^-N), and NH_4_^+^-N) concentrations. The rest of the soil was air-dried; a portion of this was used to determine the pH, while the other portion was used to determine the concentration of total carbon (TC), total N (TN), and total phosphorus (TP) which was sieved using a 0.15-mm mesh. The plant samples (shoots and roots) were dried to a constant weight at 60 °C and ground into fine powder (d < 0.15 mm), using a pulverizer (FZ102, Keheng, Shanghai, China), to determine the concentrations of TC, TN, and TP.

Approximately 5 g of fresh soil was extracted with 2 M KCl (m:v = 1:10) in a shaker, at ambient temperature for l h (200 r min^−1^), to determine the concentrations of soil DOC, NH_4_^+^-N, and NO_3_^−^-N. A portion of the extract liquid was filtered through a 0.45-μm filter then examined using a total organic carbon analyzer (Shimadzu, Toc-Vwp, Kyoto, Japan), to determine the concentration of soil DOC [32]. The other portions were run through a continuous flow analytical system (Auto Analyzer Bran + Luebbe, Hamburg, Germany) to measure the concentrations of soil NH_4_^+^-N and NO_3_^−^-N [26]. Approximately, 5 g of air-dried soil was dissolved in distilled water (m:v = 1:5), after which soil pH was determined using an acidometer (Sartorius PB-10, Gottingen, Germany). The plant (shoots and roots) and soil TC and TN contents were determined using an elemental analyzer (Elementar Vario MACRO, Elementar, Langenselbold, Germany). The concentrations of plant and soil TP were determined colorimetrically, using a spectrophotometer (Sartorius stedim biotech, Gottingen, Germany) at 880 nm after digestion with H_2_SO_4_ and HClO_4_, H_2_SO_4_, and H_2_O_2_, respectively [26]. Carbon and nutrient concentration were all expressed on a dry weight basis, and all ratios (C:N, C:P, and N:P) were expressed on a mass basis.

### 2.3. Data Statistical Analysis 

All data were tested for normal distribution before conducting a statistical analysis using Statgraphics 3.0 (STN, St Louis, MO, USA). We used one-way ANOVA (N addition rates) to analyze vegetation community characteristics (cover, species number, aboveground and belowground biomass, and R/S), vegetation (shoots and roots), and soil C, N, and P contents and their stoichiometry. We also used one-way ANOVA (plant tissues, shoots, and roots) to analyze vegetation parameters, and used another one-way ANOVA (soil sampling position, RS, and BS) to analyze the soil parameters. The least significant analysis (LSD) was used to analyze the significant difference between different factors; the significance level was set up *p* ≤ 0.05. We used Origin 2021 software (Origin Lab, Northampton, MA, USA) to analyze the correlation of data, draw figures, and express all the data in the figures and tables as mean ± standard error (SE).

## 3. Results

### 3.1. Community Characteristics 

**Figure 2:**
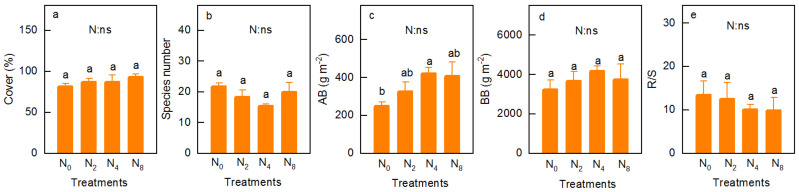
Cover (**a**), species number (**b**), AB (**c**, aboveground biomass), BB (**d**, belowground biomass) and R/S (root-shoot ratio, **e**) under the N addition rates. Different letters on the bars indicate significant difference (*p* ≤ 0.05). *p*-value of the ANOVA of N addition treatments (0, 20, 40 and 80 kg N ha^−^^1^ y^−1^) is indicated: ns, not significant.

Nitrogen addition increased the vegetation cover (*p* = 0.544, Figure 2a) and BB (*p* = 0.682, Figure 2d), slightly. However, there was a slight decrease in number of species (*p* = 0.234, Figure 2b) and R/S (*p* = 0.782, Figure 2e). The AB showed a single peak curve with increasing N addition, compared to N_0_, which was significantly increased on N_4_ (Figure 2c). Vegetation cover showed a significant line increase with increasing N addition (*R*^2^ = 0.906, *p* = 0.032, Table S1), while R/S showed a significant line decrease with increasing N addition (*R*^2^ = 0.808, *p* = 0.101, Table S1).

### 3.2. Vegetation Carbon, Nitrogen and Phosphorus

**Figure 3:**
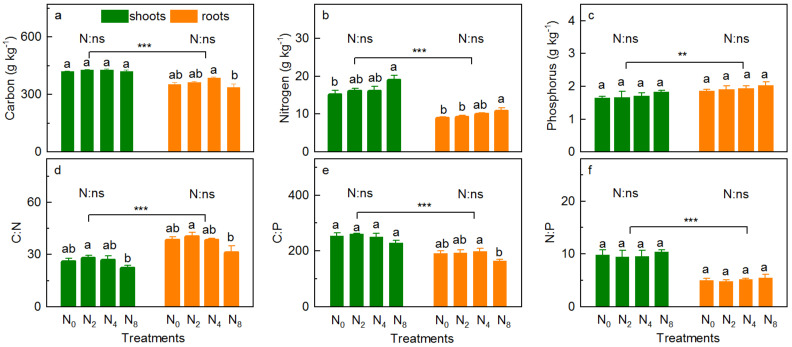
Concentrations of TC (**a**), TN (**b**) and TP (**c**), and C:N (**d**), C:P (**e**) and N:P (**f**) of plant community (shoots and roots) under the N addition rates. *p*-value of the ANOVA of N addition treatments (0, 20, 40, and 80 kg N ha^−^^1^ y^−1^) on tissues (shoots and roots) is indicated: ** *p* ≤ 0.01; *** *p* ≤ 0.001; ns, not significant.

The concentration of vegetation TC changed in the order of N_2_ > N_4_ > N_8_ > N_0_ and N_4_ > N_2_ > N_0_ > N_8_ for shoots (*p* = 0.530) and roots (*p* = 0.078) with increasing N addition, respectively (Figure 3a). However, compared with N_0_, N addition decreased the concentration of shoot TC in N_8_ slightly (Figure 3a). N addition slightly increased the concentration of TN in the shoots and roots (Figure 3b, *p* = 0.140 and 0.080, respectively) and TP (Figure 3c, *p* = 0.616 and 0.581, respectively), and showed a significant line increase with increasing N addition, respectively (*R*^2^ = 0.851, *p* = 0.050; *R*^2^ = 0.948, *p* = 0.017; *R*^2^ = 0.934, *p* = 0.022; *R*^2^ = 0.999, *p* < 0.001, Table S2). Moreover, compared with N_0_, the concentration of vegetation TN increased significantly under N_8_ (Figure 3b). N addition had no significant effect on C:N (Figure 3d, *p* = 0.191, *p* = 0.116), C:P (Figure 3e, *p* = 0.501, *p* = 0.139), and N:P (Figure 3f, *p* = 0.901, *p* = 0.690) in shoots and roots. The concentrations of shoots TC (Figure 3a, *p* < 0.001) and TN (Figure 3b, *p* < 0.001), and C:N (Figure 3e, *p* < 0.001) and N:P (Figure 3f, *p* < 0.001) were significantly higher than those in the roots, whereas the opposite was true for TP (Figure 3c, *p* = 0.003) and C:N (Figure 3d, *p* < 0.001).

### 3.3. Soil Physicochemical Property

**Figure 4:**
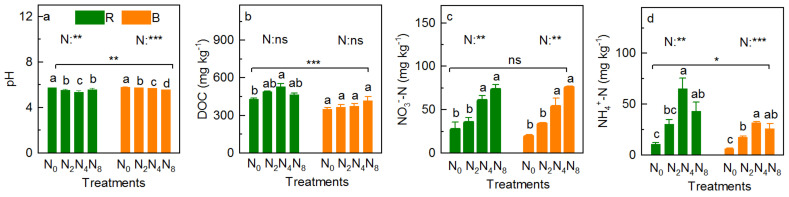
pH (**a**), DOC (**b**), NO_3_^−^-N (**c**), and NH_4_^+^-N (**d**) content of soil (rhizosphere and bulk) under the N addition rates. *p*-value of the ANOVA of N addition treatments (0, 20, 40, and 80 kg N ha^−^^1^ y^−1^) on soil compartment (rhizosphere and bulk soils) is indicated: * *p* ≤ 0.05; ** *p* ≤ 0.01; *** *p* ≤ 0.001; ns, not significant.

Nitrogen addition reduced the value of soil pH (*p* = 0.002 and *p* < 0.001 for RS and BS, respectively), significantly; regarding the RS, soil pH was in the order of N_0_ > N_8_ > N_2_ > N_4_, while the value was reduced with increasing N addition (Figure 4a, *R*^2^ = 0.03, *p* = 0.033, Table S3). N addition slightly increased the soil DOC content (*p* = 0.098 and 0.222 for RS and BS, respectively), which was in the order of N_4_ > N_2_ > N_8_ > N_0_ for RS and increased with increasing N addition in BS (Figure 4b, *R*^2^ = 0.959, *p* = 0.014, Table S3). N addition increased the concentration of NO_3_^−^-N (Figure 4c, *p* = 0.002 for RS and BS) and NH_4_^+^-N (Figure 4d, *p* = 0.004 and 0.001 for RS and BS, respectively) significantly; the concentration of soil NO_3_^−^-N increased with increasing N addition (*R*^2^ = 0.889, *p* = 0.038, *R*^2^ = 0.977, *p* = 0.008, respectively, Table S3). However, the concentration of NH_4_^+^-N was in the order N_4_ > N_8_ > N_2_ > N_0_ (Figure 4d).

**Figure 5:**
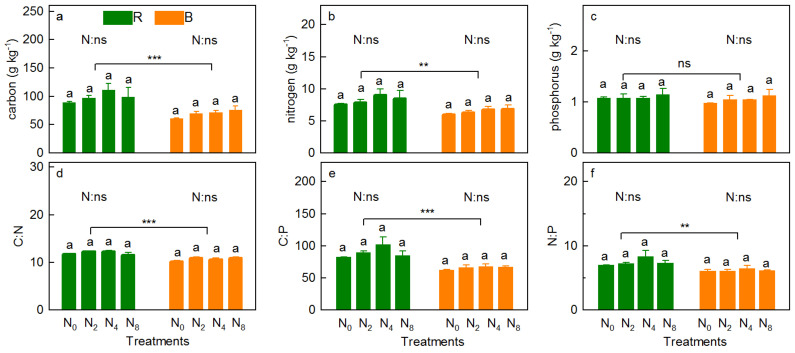
Concentrations of TC (**a**), TN (**b**), and TP (**c**), and C:N (**d**), C:P (**e**), and N:P (**f**) of soil (rhizosphere and bulk) under the N addition rates. *p*-value of the ANOVA of N addition treatments (0, 20, 40, and 80 kg N ha^−^^1^ y^−1^) on soil compartment (rhizosphere and bulk soil) is indicated: ** *p* ≤ 0.01; *** *p* ≤ 0.001; ns, not significant.

Nitrogen addition slightly increased the concentration of soil TC (Figure 5a, *p* = 0.530 and 0.246 for RS and BS, respectively), TN (Figure 5b, *p* = 0.638 and 0.447 for RS and BS, respectively) and TP (Figure 5c, *p* = 0.909 and 0.578 for RS and BS, respectively), and C:P (Figure 5e, *p* = 0.098 and 0.222 for RS and BS, respectively), C:N for BS (Figure 5d, *p* = 0.098), and N:P for RS (Figure 5f, *p* = 0.222). Specifically, the TC and TN concentrations were all in the order of N_4_ > N_8_ > N_2_ > N_0_ for RS, while they slightly increased with increasing N addition rates for BS (*p* = 0.087 and *p* = 0.075, respectively; Table S3). Moreover, the concentration of TP ranged from 1.08 to 1.18, 0.98 to 1.14 g kg^−1^ for RS and BS, respectively (Figure 5c). The RS pH was significantly lower than that of BS (Figure 4a, *p* < 0.001), whereas the concentrations of DOC (Figure 4b, *p* < 0.001), NH_4_^+^-N (Figure 4d, *p* = 0.029), TC (Figure 5a, *p* < 0.001), TN (Figure 5b, *p* = 0.004), C:N (Figure 5d, *p* < 0.001), C:P (Figure 5e, *p* < 0.001), and N:P (Figure 5f, *p* = 0.003) in RS were significantly higher than those in BS, but not in NO_3_^−^-N (Figure 4c, *p* = 0.701) and TP (Figure 5c, *p* = 0.292).

### 3.4. Correlation between Soil and Vegetation

**Figure 6:**
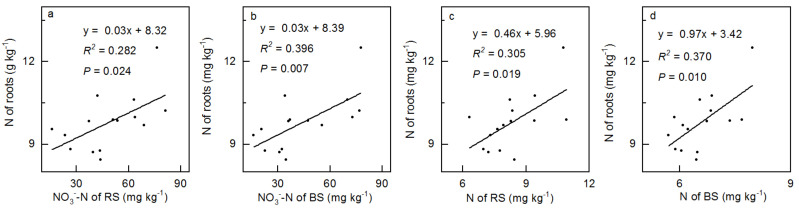
Correlations between soil (rhizosphere and bulk, x) and roots (y) nutrients. Correlation of rhizosphere soil NO_3_^−^-N and roots N (**a**), bulk soil NO_3_^−^-N and roots N (**b**), rhizosphere soil N and roots N (**c**) and bulk soil N and roots N (**d**).

The concentrations of soil NO_3_^−^-N and N were closely correlated with the root TN (Figure 6, Table S4). Following the increase in N addition, the concentration of roots TN had a significant and positive correlation with NO_3_^−^-N in the rhizosphere (Figure 6a, *R*^2^ = 0.282, *p* = 0.024) and bulk soil (Figure 6b, *R*^2^ = 0.396, *p* = 0.007), respectively. The concentration of roots TN and TN in the rhizosphere (Figure 6c, *R*^2^ = 0.305, *p* = 0.019) and bulk soil (Figure 6d, *R*^2^ = 0.370, *p* = 0.010) showed a similar tendency. There was no significant correlation between the C:N, C:P, and N:P of vegetation and soil (Table S5).

## 4. Discussion

### 4.1. Effects of N Addition on Community Characteristics

Previous studies show that N addition has profound effects on biodiversity [1,6,7,10,33,34]. These studies assert that aboveground light competition [1,7], soil acidification [1,7,34,35], and soil ammonium toxicity [1,34] are the main causes of the loss of plant species. Specifically, N addition improves the AB of the plant community, which causes a switch of competition, from soil N to light competition [1,7], and inhibits the growth of vulnerable or rare species, which could even become extinct [6,10]. Furthermore, the imbalance of cationic ions such as manganese (Mn^2+^), caused by soil acidification inhibits the photosynthetic rate and growth of plants [34], thus excluding some non-acid-tolerant species [33]. Additionally, plant root growth directly inhibits and even causes plant death under high NH_4_^+^ concentration in soil [1,34]. The slight decrease in the number of species noted in this study (Figure 2b) may be mainly due to the relatively short experimental time (only 10 times during 2-year), which was not enough to make the loss of species noticeable [10]. Additionally, some heliophile species located at the bottom were lost owing to the relatively high vegetation cover, before the experiment began, which attenuated the effects of the loss of species owing to N addition. Compared with N_0_, N addition slightly increased community cover (Figure 2a) and biomass (including AB and BB, Figure 2c,d), which was a result of the N addition alleviating the N limitation, owing to slow N mineralization rates [6,10,28]. Generally, the response of biology to N addition is a nonlinear relationship, in other words, when N addition exceeds the critical threshold, biology will affect the function of ecosystems in unpredictable ways [10,36]. In this study, the AB and BB of the alpine meadow presented a single-peak curve change pattern with an increase in N addition (the highest value in the N4 treatment, Figure 2c,d), which may be attributed to the fact that the low level of N addition alleviated the limitation of N [6,10], while the high N (N_8_) reduced the photosynthesis of plants (the low TC concentration in N_8_ was circumstantial evidence), inhibited plant growth, and reduced the biomass in the vegetation [15,37]. The R/S reflects the allocation of plants to resources [38,39]. Our results further showed that N addition slightly decreased the R/S of the alpine meadow (Figure 2e), which mainly depends on that N addition improves AB of the plant community, which causes significant light competition [1,7]. The increase in photosynthate distribution above the ground resulted in a decrease in the R/S. In other words, the decrease in R/S in this study is mainly attributed to the fact that the increase in AB (Figure 2c, ranging from 30 to 67%) is higher than that of BB (Figure 2d, ranging from 13 to 28%).

### 4.2. Effects of N Addition on Vegetation TC, TN and TP Content

Previous studies show that N addition alters the dynamics of plant TC [1,11,40] and nutrients [2,12]. These studies indicate that N addition directly increases the concentration of available N in the soil and alleviates the N limitation in this meadow [6,10], which increases carbon dioxide (CO_2_) uptake via the photorespiration pathway [40] and further promotes photosynthesis in plants [10,37]. The slight increase in vegetation TC (except for roots in N_8_ plots) noted in this study (Figure 3a) may be explained by the above reason. However, under the treatment of N_8_, the concentration of vegetation TC decreased slightly (Figure 3a), as a result of the high concentration of N, which is harmful to the growth of plants and reduces the photosynthetic capacity of plants [37]. Additionally, N addition increased the concentration of vegetation N (Figure 3b) as it directly increases the concentration of available N in the soil (Figure 4c,d), which alleviated the N limitation in this meadow [6,10] by increasing the N absorption of available N by plant under N addition [13], especially in N_8_ (Figure 3b). Furthermore, N and P synergistically interact in plant metabolism; N addition could also improve the concentration of plant TP [3,6,12,41], which is consistent with our results (Figure 3c). N addition enhances the plant acquisition of TP for the following reasons: (1) N addition in the form of NH_4_^+^ may increase the availability of soil P by decreasing soil pH [3,12]; (2) N addition increases the plant uptake of soil P by stimulating the activity of root phosphomonoesterase and acid phosphatase [12,22,41], and increases the mycorrhizal fungi efficiency of P uptake by increasing C allocation [3]; and (3) the biological N:P needs to be maintained in a stable state [6]; the plants increase the absorption of P to maintain a stable N:P [12,41]. However, the increase in sight of the vegetation TP (Figure 3c) may partly be attributed to the fact that low N addition is not sufficient to significantly change its dynamics. 

The proportional relationship of vegetation elements depends on the specific response of various elements to N addition [42]. In this study, the response of vegetation (shoots and roots) C:N, C:P, and N:P showed complex changes (increase or decrease) with different intensities of N addition (Figure 3d–f). N addition significantly increased available N content in the soil (Figure 4c,d), hence increasing the N content of the vegetation (Figure 3b) through absorption [13]. Simultaneously, N addition also changes the C allocation of vegetation [39], for instance, the concentration of vegetation C was decreased under N_8_. These two factors cause N addition to decrease the C:N (Figure 3d,e) and C:P ratios. The small variation in the N:P ratio is mainly attributed to the variety of P-acquisition pathways that were activated to remise the P deficiency caused by N deposition [3].

### 4.3. Effects of N Addition on Physicochemical Property

Soil acidification is one of the direct responses of soil to N addition [14,29,30]. Lower soil pH leads to the leaching loss of the available nutrients in soil and the enrichment of cationic ions such as Mn^2+^ and ions (Fe^3+^), this further affects the growth of plants [34,35]. In this study, N addition significantly decreased the soil pH (Figure 4a) for different reasons. The decrease in the RS pH may be attributed to a change in root excretion, caused by N deposition [43]. Specifically, NH_4_Cl was used to simulate N deposition, after which proton (H^+^) would be generated when NH_4_^+^ was absorbed by plant roots [44]; however, following the increase in N addition, the meadow ecosystem reaches N saturation (under N_8_), and the increased N input would make the whole ecosystem appear to have N surplus [18]. Further, the absorption of NH_4_^+^ through the roots is reduced, which results in an increase in soil pH. However, the NH_4_^+^-N in BS is nitrated into nitrate (NO_3_^−^) and the generated H^+^ and NO_3_^−^ are further easily lost through runoff and leaching, with a large number of cation nutrients (such as potassium, calcium, and magnesium) [13,14]. These changes resulted in significant reduction in BS pH (Figure 4a). The rapid decrease in BS pH in response to N addition suggests that the capacity of BS to resist soil acidification in this study is very limited [11].

DOC is an important part of the C and N cycles in meadow soil [32], and its dynamics depend on biodegradation, oxidation, and soil physicochemical absorption and release [5], essentially on the balance between the source and consumption. Previous studies show that N addition increases the concentration of soil DOC [45], which is consistent with the results of this study (Figure 4b). This may be attributed to the fact that N addition increased the input of soil organic residues such as litterfall, root sloughing, root exudate [4,14], and water soluble substances [46], which stimulate the production and release of soil DOC [5]. However, the decrease in RS DOC content N_8_ (compared with N_4_) may be attributed to the reduction in litterfall (Figure 2c), roots, and microbes under high-level N input owing to soil acidification and the loss of cation nutrients [13,14].

Soil NO_3_^−^-N and NH_4_^+^-N are both the main forms of soil N, which are directly used by plants, and mainly obtained from the mineralization of organic N [13]. Previous studies indicated that N addition directly or indirectly increases the concentration of NO_3_^−^-N and NH_4_^+^-N in the soil [1,13,47], which is consistent with the same results in this study (Figure 4c,d). There were both similar and different reasons for this. Similarly, N addition not only increased the biomass of community (Figure 2c), which is the raw material of soil NO_3_^−^-N and NH_4_^+^-N, but also stimulated soil microbial communities, and increased the rates of nitrification and N mineralization, hence increasing the N available in the soil [13,47]. The different reasons are as follows: regarding NO_3_^−^-N, the external N input increased nitrification, as the availability of soil NH_4_^+^ is one of the most important factors in determining the soil nitrification rate [15], and further increased the concentration of NO_3_^−^-N (Figure 4c); regarding NH_4_^+^-N, (1) NH_4_Cl was used to provide exogenous N which would directly increase the concentration of soil NH_4_^+^-N, (2) NH_4_Cl addition in this study accelerated the growth of heterotrophic bacteria with N fixation, that is, N addition is beneficial to heterotrophic bacteria and is advantageous in the competition for NH_4_^+^-N [13,34]. The concentration of soil NH_4_^+^-N showed a unimodal curve with N addition rates, especially in the RS (Figure 4d). This is mainly because the alpine meadow was mainly restricted by N. However, its N saturation threshold was approximately 50 kg N ha^−1^ y^−1^ [6,10]; when the N addition level exceeds this value, the excess NH_4_^+^ will cause soil nitrification [48] and then reduce the concentration of soil NH_4_^+^-N. A significant increase was found in the soil NO_3_^−^-N (Figure 4c) and NH_4_^+^-N (Figure 4d); unfortunately, there were no significant effects on plant biomass (Figure 2c,d), R/S (Figure 2e), plant C and nutrients, and their stoichiometry (Figure 3). These results imply that the significant increase in the available N caused by N addition is not sufficient to make a marked difference in the plant properties, and nutrient limitation on the N limitation alpine meadow of QTP.

Previous studies indicate that N addition increases the concentration of soil TC and TN, mainly for the following reasons: (1) The increase in community biomass due to N addition, provides adequate raw material for increasing soil TC and TN; (2) N addition increases the soil mineralization rate [13], which could decompose the organic matter produced by the increased biomass in time. Unfortunately, we found no significant effects of N addition on the concentrations of TC and TN in soil (Figure 5a,b). This may be attributed to the following: (1) The N addition rates imposed in this study are relatively low compared to those in previous studies on N additions in the same areas (Hongyuan County) [8], on the northeastern margin of the QTP; (2) there is a lot of evidence that long-term N addition could change the soil properties [9,11], but the N addition durations in this study were done only 10 times during the 2-year period, which is too short for soil TC, TN, and TP dynamics to change significantly. There is need for further research to elucidate the response and mechanism of soil properties to long-term low-level N addition. Soil TP mainly comes from the weathering of rocks, only a small portion is directly from organic matter decomposition [49,50]. Moreover, the concentration of TP in soil is stable under N addition (Figure 5c). The ratio of soil C:N, C:P, and N:P mainly depends on the imbalanced changes in soil C, N, and P content under different N addition rates [23]. The relatively stable soil C:N, C:P, and N:P ratios suggest that the soil C and nutrient cycling cannot be significantly changed by the short-term low-N addition in this study.

### 4.4. The Linkage between Plant and Soil

As two important relatively independent parts of terrestrial ecosystems, a series of material circulation and energy flows exist between vegetation and soil to ensure the dynamics and functions of ecosystems [18,26]. For example, plants absorb nutrients such as N and P from soil in the form of available nutrients, and transform them into important compounds (e.g., proteins, nucleic acids, and hormones) [51,52]. Some compounds are retained in vegetation as biomass, while others are returned to the soil in the form of litter, where they further decompose and are converted to nutrients in the soil, which are reabsorbed by plants [51,52]. However, the rhizosphere plays an important role in the interaction between plants and soil and is an important channel for the interaction between the two [26]. A previous study shows that N addition not only affects soil properties indirectly, but also affects the interactions between plants and soil [34]. In the alpine meadow of the QTP, the TN and TP contents in the soil are very rich [28]. However, the part that can be absorbed by plants is very limited as the low temperature limits the mineralization of the soil [28]. We found a significantly positive correlation between the concentration of soil (rhizosphere and bulk) NO_3_^−^-N and TN, and root TN, respectively (Figure 6), suggesting that the root is more closely related to the shoot, and RS is easier than BS in the formation of a linkage between vegetation and soil under N addition. From the perspective of plants, roots are not only important organs for plants to directly absorb nutrients from the soil, but also the most sensitive organs for ascertaining any changes in the soil [53,54], which easily absorb enough N for plant growth after N addition, to form a balance between plant and soil N (Figure 6). From the perspective of soil, the concentration of plant N mainly depends on the availability of soil N [55], and the availability of N in alpine meadow soil is very low under natural conditions [28]. However, this increases the soil available N concentration under N addition (Figure 4c,d), which significantly alleviates N limitation in the alpine meadow [6,10] and balances the N content of plants and soil (Figure 6).

Many related studies show that the stoichiometry of plants and soils is obviously correlated in many terrestrial ecosystems [18,22,23,24], such as *Robinia pseudoacacia* [23] and Eucalyptus plantations [18]. Unfortunately, we did not find any significant correlation between plant and soil stoichiometry (Table S5), which mainly depends on the soil stoichiometry, which was controlled by plant function group identity rather than the availability of soil elements, as each plant has a unique internal nutrient regulation mechanism [56]. This study also suggests that there is a highly complex relationship between soil and plant C, N, and P stoichiometry (Figure 6 and Table S5) [19]. Additionally, the significant difference in plant stoichiometry at different growth stages [57] may be another reason for the insignificant correlation between the stoichiometry of plant and soil systems. Therefore, further studies need to pay more attention on under what conditions does the stoichiometry of vegetation-soil system become coupled or uncoupled, and sampling is necessary at different points [57].

### 4.5. Limitations

This study has some limitations. Previous studies indicated that soil properties, especially in soil microbes, have high temporal dynamics [22,57]. Further, the results were shown based on only one sampling time point, which may not reflect the whole process sufficiently; further studies should be conducted at different time points to ascertain the response of vegetation-soil under N addition. Moreover, previous studies also concluded that the duration of time affects the response to N addition significantly [9,11]. Further, a long-term observation platform is necessary, to comprehensively study the variation of vegetation-soil under N addition.

## 5. Conclusions

This study explores the responses of plant communities, vegetation TC, TN, and TP, soil properties, and the linkage between vegetation TC, TN, and TP, and soil DOC, NH_4_^+^-N, NO_3_^−^-N, TC, TN, and TP under different N addition rates after 10 N additions in the alpine meadow in the QTP. Our results show that (i) N addition significantly increased and decreased the soil NO_3_^−^-N and NH_4_^+^-N, and the soil pH, respectively, slightly increasing the vegetation cover, aboveground and belowground biomass, and the concentration of vegetation TC (except for roots in N_8_ plots), TN and TP; and soil DOC, TC, TN, TP; and C:N, C:P, and N:P ratios; it however slightly decreased the number of species and R/S. (ii) There were significant relationships between soil NO_3_^−^-N and N, and root N, but there was no significant correlation between plant and soil stoichiometry under different N addition rates. The results suggest that short-term low-N addition insignificantly affects the plant communities, vegetation, and soil TC, TN, TP, and their stoichiometry, and that there was no strong correlation between plant and soil TC, TN, and TP, and their stoichiometry. Therefore, more long-term observation platforms and all-round monitoring are required, to ascertain the response patterns of vegetation-soil ecosystem C and nutrient cycling under N addition, comprehensively and accurately.

## Figures and Tables

**Table 1:** The study design.

Treatments	N Addition Rates (kg ha^−1^ y^−1^)	NH_4_Cl Used Amount (kg ha^−1^ y^−1^)
N_0_	0	0
N_2_	20	76.43
N_4_	40	152.86
N_8_	80	305.71

## Data Availability

The datasets used and/or analyzed during the current study are available from the corresponding author upon reasonable request—Zhen’an Yang.

## Supplementary Materials

The following are available online at https://www.mdpi.com/article/10.3390/ijerph182010998/s1. Table S1: Correlations between nitrogen deposition rates (x) and community characteristics (y). Table S2: Correlations between nitrogen deposition rates (x) and vegetation TC, TN, and TP (shoots and roots, y). Table S3: Correlations between nitrogen deposition rates (x) and soil property (y). Table S4: Correlations between soil property (rhizosphere and bulk, x) and vegetation TC, TN, and TP (shoots and roots, y). Table S5: Correlations between soil (rhizosphere and bulk, x) and vegetation C:N, C:N, and N:P (shoots and roots, y).
