# Supplementary material for: Effect of Short-Term Low-Nitrogen Addition on Carbon, Nitrogen and Phosphorus of Vegetation-Soil in Alpine Meadow
Source: Int J Environ Res Public Health. 2021 Oct 19;18(20):10998. doi: 10.3390/ijerph182010998 (PMC8536122; doi:10.3390/ijerph182010998)
Supplement: Supplementary file 1 [file ijerph-18-10998-s001.zip › ijerph-1381148-suppl.pdf]

Table S1. Correlations between nitrogen deposition rates (x) and community characteristics (y).

	Cover (%)	Species number	Aboveground biomass (g m^{-2})	Belowground biomass (g m-2)	R/S
N addition rates	**R^2= 0.906**	R^2= 0.963	R^2= 0.903	R^2= 0.856	R^2= 0.824
	**P = 0.032**	P = 0.111	P = 0.180	P = 0.219	P = 0.061

Table S2. Correlations between nitrogen deposition rates (x) and vegetation TC, TN and TP (shoots and roots, y)

		TC	TN	TP
N addition rates	Shoots	R^2= 0.753	**R^2= 0.851**	**R^2= 0.934**
		P = 0.287	**P = 0.050**	**P = 0.022**
	Roots	R^2= 0.639	**R^2= 0.948**	**R^2= 0.999**
		P = 0.347	**P = 0.017**	**P < 0.001**

Table S3. Correlations between nitrogen deposition rates (x) and soil property (y)

		pH	DOC	NO_3^--N	NH_4^+-N	TC	TN	TP
N addition rates	Rhizosphere	R^2= 0.451	R^2= 0.961	**R^2= 0.889**	R^2= 0.675	R^2= 0.605	R^2= 0.416	R^2= 0.942
		P = 0.428	P = 0.113	**P = 0.038**	P = 0.330	P = 0.363	P = 0.441	P = 0.140
	Bulk	**R^2= 0.903**	**R^2= 0.959**	**R^2= 0.977**	R^2= 0.878	R^2= 0.749	R^2= 0.784	R^2= 0.850
		**P = 0.033**	**P = 0.014**	**P = 0.008**	P = 0.202	P = 0.087	P = 0.075	P = 0.051

Table S4. Correlations between soil property (rhizosphere and bulk, x) and vegetation TC, TN and TP (shoots and roots, y).

		Shoots			Roots		
		**TC**	**TN**	**TP**	**TC**	**TN**	**TP**
**Rhizosphere soil**	**DOC**	R^2= -0.070			R^2= -0.070		
		P = 0.772			P = 0.760		
	**TC**	R^2 = 0.031			R^2= -0.041		
		P = 0.251			P = 0.514		
	**NO_3^--N**		R^2= 0.071			**R^2= 0.282**	
			P = 0.174			**P = 0.024**	
	**NH_4^+-N**		R^2= -0.076			R^2= 0.006	
			P = 0.903			P = 0.318	
	**TN**		R^2= -0.047			**R^2 = 0.305**	
			P = 0.551			**P = 0.019**	
	**TP**			R^2 = -0.075			R^2= -0.058
				P = 0.866			P = 0.644
**Bulk soil**	**DOC**	R^2 = 0.062			R^2 = 0.068		
		P = 0.188			P = 0.177		
	**TC**	R^2= 0.150			R^2= 0.041		
		P = 0.086			P = 0.228		
	**NO_3^--N**		R^2= 0.115			**R^2= 0.396**	
			P = 0.117			**P = 0.007**	
	**NH_4^+-N**		R^2= -0.118			R^2= -0.103	
			P = 0.769			P = 0.715	
	**TN**		R^2= -0.014			**R^2= 0.370**	
			P = 0.386			**P = 0.010**	
	**TP**			R^2 = -0.067			R^2= -0.067
				P = 0.738			P = 0.737

Table S5. Correlations between soil (rhizosphere and bulk, x) and vegetation C:N, C:N and N:P (shoots and roots, y).

		Shoots			Roots		
		**C:N**	**C:P**	**N:P**	**C:N**	**C:P**	**N:P**
**Rhizosphere soil**	**C:N**	R^2= 0.579<br>P = 0.152			R^2= -0.463<br>P = 0.844		
	**C:P**		R^2= -0.443<br>P = 0.804			R^2= -0.008<br>P = 0.422	
	**N:P**			R^2 = -0.257<br>P = 0.597			R^2= -0.377<br>P = 0.713
**Bulk soil**	**C:N**	R^2= 0.383<br>P = 0.233			R^2 = -0.383<br>P = 0.721		
	**NO_3^--N**		R^2= -0.376<br>P = 0.713			R^2= -0.493<br>P = 0.933	
	**N:P**			R^2 = -0.386<br>P = 0.724			R^2= -0.286<br>P = 0.622
